# NOD Signaling and Cell Death

**DOI:** 10.3389/fcell.2019.00208

**Published:** 2019-10-02

**Authors:** Valentin J. Heim, Che A. Stafford, Ueli Nachbur

**Affiliations:** ^1^The Walter and Eliza Hall Institute of Medical Research, Parkville, VIC, Australia; ^2^Department of Medical Biology, University of Melbourne, Melbourne, VIC, Australia; ^3^Gene Center and Department of Biochemistry, Ludwig-Maximilians-Universität München, Munich, Germany

**Keywords:** RIPK2, NOD2, ubiquitin, inflammation, cell signaling

## Abstract

Innate immune signaling and programmed cell death are intimately linked, and many signaling pathways can regulate and induce both, transcription of inflammatory mediators or autonomous cell death. The best-characterized examples for these dual outcomes are members of the TNF superfamily, the inflammasome receptors, and the toll-like receptors. Signaling via the intracellular peptidoglycan receptors NOD1 and NOD2, however, does not appear to follow this trend, despite involving signaling proteins, or proteins with domains that are linked to programmed cell death, such as RIP kinases, inhibitors of apoptosis (IAP) proteins or the CARD domains on NOD1/2. To better understand the connections between NOD signaling and cell death induction, we here review the latest findings on the molecular regulation of signaling downstream of the NOD receptors and explore the links between this immune signaling pathway and the regulation of cell death.

## Activation of the NOD Pathway

### Pattern Recognition Receptors

Sensing of pathogen-associated molecular patterns (PAMPs) is the initiating step in an efficient immune reaction to a bacterial, viral or parasitic threat. The intracellular receptors nucleotide-binding oligomerization domain-containing protein 1 and 2, NOD1 and NOD2, are members of the pattern recognition receptors (PRR) and recognize intracellular bacterial peptidoglycans. The PRR family consists of a range of cytoplasmic or transmembrane stress sensors that recognize PAMPs and damage-associated molecular patterns (DAMPs).

**FIGURE 1:**
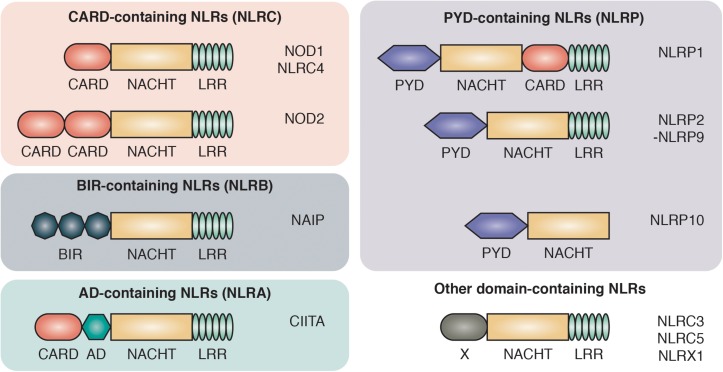
Domain architecture of NOD-like receptors. NLRs are composed of C-terminal leucine-rich repeats (LRR), an intermediate nucleotide-binding-domain (NACHT) and variable N-terminal protein-protein interaction domains that divide NLRs into different protein subfamilies: NLRCs contain one to two caspase activation and recruitment domains (CARD). NLRBs contain multiple baculovirus inhibitor of apoptosis protein repeats (BIR). NLRAs contain at least one acidic transactivating domain (AD) and the NLRP subfamily harbors a pyrin domain (PYD).

PRRs are divided into two main groups based on their cellular localization: the transmembrane/endosome-associated PRRs, consisting of toll-like receptors (TLRs) and C-type Lectin receptors, and the cytosolic PRRs which are further divided into the RIG-1-like receptors, AIM2-like receptors and the NOD-like receptors (NLRs) (Bertin et al., 1999; Inohara et al., 1999; Ogura et al., 2001b). NLRs are characterized by a central 300–400 amino acid long NACHT domain (also referred to as the NOD or NBD domain) that has predicted nucleoside-triphosphatase activity and facilitates its oligomerization. On the C-terminus, NLRs bear multiple leucine-rich repeats (LRRs) that mediate ligand sensing (Figure 1).

The NLRs consists of four subfamilies, based on the nature of their N-terminal effector domain: The NLRC subfamily is characterized by one or multiple N-terminal caspase activation and recruitment domains (CARDs) that allow direct interaction with other CARD-containing proteins. Among the NLRCs, NOD1 and NOD2 represent the two best characterized members and are sensors of intracellular bacterial peptidoglycan (Girardin et al., 2003a, b). The NOD1 and NOD2 pathways have been associated with a range of autoimmune disorders, most prominently with inflammatory bowel disease (IBD). Single nucleotide polymorphisms (SNPs) in the NOD2 gene were the first identified genetic risk factors associated with Crohn’s disease (CD) (Hugot et al., 2001; Ogura et al., 2001a).

The second key member of the NLR family are the NLRPs, which are best known for their role in the formation of large oligomeric complexes, the inflammasomes. Inflammasomes mediate the processing, activation and secretion of pro-inflammatory cytokines as well as the induction of pyroptosis through the recruitment and activation of caspase-1 (Martinon et al., 2009). NLRPs contain an N-terminal pyrin domain (PYD) that is also known as a “death fold,” which is evolutionary related to the death domain (DD) found in cell death-inducing receptors including Fas, TNFR1 and TRAIL R-1 and R-2 (Fairbrother et al., 2001). The two smaller subfamilies of NLRs are NLRA and NLRB. The NLRA (A for acidic transactivating domain) subfamily only includes one member, class II major histocompatibility complex transactivator (CIITA), that serves as an activator of MHC class II antigen presentation (Nickerson et al., 2001). Members of the NLRBs [B for baculovirus inhibitor of apoptosis protein repeat (BIR)] have one or multiple N-terminal BIR domains. The approximately 70 amino acid zinc-binding BIR domain was first identified through sequence homology among proteins belonging to the Inhibitors of Apoptosis (IAP) family and is mostly recognized for its role in promoting cell survival (Silke and Meier, 2013).

### Expression of NOD Receptors

NOD1 and NOD2 both recognize building blocks of bacterial peptidoglycans and share identical downstream signaling pathways. One important difference between these two PRRs is their distinct expression pattern: NOD1 is broadly expressed in a variety of cells including epithelial cells, stromal cells and endothelial cells (Inohara et al., 1999; Park et al., 2007b). In contrast, the expression of NOD2 is more restricted and highest in the hematopoietic compartment, most prominently in cells of myeloid origin such as monocytes (Ogura et al., 2001b), dendritic cells and macrophages (Pashenkov et al., 2010). Furthermore, expression of NOD2 has also been demonstrated in hematopoietic cells of lymphoid origin including B cells (Petterson et al., 2011), CD4^+^ and CD8^+^ T cells (Caetano et al., 2011; Lin et al., 2013) and regulatory T cells (Kerns et al., 2009). Notably, NOD2 is also expressed by various epithelial cell types, particularly in Paneth cells located within the ileum of the intestinal tract (Uehara et al., 2007).

Basal expression levels of NOD1 and NOD2 are generally low but can be induced by various immunomodulators. In intestinal epithelial cells, interferon-gamma (IFN-γ) (Rosenstiel et al., 2003), tumor necrosis factor-alpha (TNF-α), lipopolysaccharide (LPS) (Kim Y.G. et al., 2008), 1,25-dihydroxycholecalciferol (Wang et al., 2010), and butyrate (Leung et al., 2009) have been shown to induce the upregulation of NOD2 mRNA. Additionally, we and others observed that IFN-γ increases NOD2 protein levels in bone marrow-derived macrophages and is required for an effective cytokine response after stimulation with the NOD2 ligand muramyl dipeptide (MDP) (Nachbur et al., 2015; Fekete et al., 2017; Stafford et al., 2018).

Once expressed, NOD1 and NOD2 reside in the cytosol and localize to bacterial entry sites at the plasma membrane (Barnich et al., 2005; Kufer et al., 2008). Both receptors constantly interact with the actin cytoskeleton, which facilitates their rapid relocalization upon stimulation (Legrand-Poels et al., 2007). More recent studies indicate that NOD1 and NOD2 are associated with early endosomes that serve as assembly platforms for NOD signaling complexes (Irving et al., 2014; Nakamura et al., 2014).

Expression levels of NOD1 and NOD2 are held in check through constant degradation by the proteasome. This is counteracted by several chaperones including HSP90, SGT1 (Correia et al., 2007; Mayor et al., 2007; Lee et al., 2012) and HSP70 (Mohanan and Grimes, 2014) which bind and stabilize NOD proteins. Their importance for NOD signaling has been demonstrated using small molecule inhibitors that decrease NOD2 ligand-dependent activation.

A candidate E3 ubiquitin ligase that was shown to ubiquitinate NOD2 and target it for proteasomal degradation is TRIM27 (Zurek et al., 2012). NOD2 was shown to be modified with K48-linked ubiquitin chains after overexpression of TRIM27 in HEK293T cells while siRNA-mediated knockdown of TRIM27 led to the stabilization of NOD2 protein levels. Recently, NLRP12 was shown to indirectly regulate NOD2 protein levels in monocytes. NLRP12 activation leads to the sequestering of HSP90, which in turn promotes K48-linked ubiquitination and degradation of NOD2 in response to MDP. The significance of NLRP12 as a regulator of NOD2 signaling was highlighted by the finding that LPS-primed NLRP12-deficient mice are highly susceptible to secondary challenge by bacterial MDP (Normand et al., 2018).

### Canonical Activation of NOD1 and NOD2

Upon its discovery, various groups reported that NOD1 is activated by lipopolysaccharides (LPS) and mediates the activation of NF-κB in a MyD88-independent manner (Girardin et al., 2001; Inohara et al., 2001; Kobayashi et al., 2002). However, through the use of ultra-pure LPS, and synthetic NOD ligands, it has become clear that NOD1 and NOD2 sense distinct monomeric peptidoglycan (PGN) fragments: NOD1 is activated by γ-D-glutamyl-meso-diaminopimelic acid (DAP), a PGN fragment that is present in the cell wall of all Gram-negative and certain Gram-positive bacteria (Chamaillard et al., 2003; Girardin et al., 2003a). NOD2 recognizes muramyl dipeptide (MDP), a PGN fragment found in both Gram-negative and Gram-positive bacteria (Girardin et al., 2003b; Inohara et al., 2003).

Multiple mechanisms of how MDP and DAP are transported into the cytosol to activate NOD1/2 have been reported. In agreement with a role of NODs as sensors of intracellular bacterial infections, NOD1 activation during infection has first been reported with the facultatively intracellular pathogen *Shigella flexneri* (Girardin et al., 2001). Moreover, extracellular DAP can be delivered to the cytosol by type III and IV secretion systems, for instance in *Helicobacter pylori* (Viala et al., 2004), or through bacterial outer membrane vesicles (OMVs). OMVs are small secreted vesicles derived from the outer membrane of Gram-negative bacteria that are able to penetrate the intestinal mucus layer and interact with epithelial cells (Sanchez et al., 2010). Only recently it has been shown that OMVs from probiotic and commensal strains of *Escherichia coli* can be endocytosed by intestinal epithelial cells where they colocalize with NOD1 and trigger its aggregation. OMVs are therefore important contributors to the maintenance of the intestinal homeostasis (Canas et al., 2018). On the other hand, there is substantial findings that OMVs from pathogenic bacteria contribute to their virulence, for instance of *Neisseria gonorrhoeae*, *Pseudomonas aeruginosa* (Kaparakis et al., 2010), *Salmonella enterica* (Yoon et al., 2011), *Brucella abortus* (Pollak et al., 2012), and *Legionella pneumophilia* (Jager et al., 2015; Jung et al., 2016).

Further mechanisms of how NOD ligands translocate to their intracellular receptors include passive transport through oligopeptide transporters such as SLC15A1 (Vavricka et al., 2004; Ismair et al., 2006), or active transport processes such as phagocytosis of live or fragmented bacteria or through epithelial junction transfer (Kasper et al., 2010). In accordance with the localization of NODs to endosomes, endocytosis is another important entry route for NOD ligands (Lee et al., 2009; Marina-Garcia et al., 2009). Certain cell types, in particular dendritic cells express the endosomal peptide transporters SLC15A3 and SLC15A4, that facilitate this process (Nakamura et al., 2014).

Once in the cytoplasm, PGN binds to NOD1/2 and induces a downstream signaling cascade resulting in the induction of a transcriptional response. *In silico* modeling of human and zebrafish NOD2 indicated a hydrophobic pocket on the concave face of the LRR as a putative binding site of MDP to NOD2 (Tanabe et al., 2004; Maharana et al., 2014). This was validated using information gathered from the rabbit NOD2 crystal structure, where mutating amino acids within the hydrophobic core of the LRR reduced, respectively abolished MDP-dependent NF-κB activation (Maekawa et al., 2016). Using surface plasmon resonance (SPR), Grimes et al. provided the first biochemical evidence that MDP bound directly to NOD2 with a relatively high affinity (KD = 51 nM) (Grimes et al., 2012). Interestingly, the affinity of MDP to NOD2 was pH-dependent and highest in the pH range from 5.0 to 6.5. This data suggests that *in vivo* binding could occur in an acidic cellular compartment, for instance in endosomes. Due to the high degree in sequence homology, ligand binding of NOD1 is believed to occur in a similar manner, however a crystal structure that could confirm this theory is still missing. Nevertheless, direct interactions between the NOD1 LRR domain and several agonists, such as TriDAP (L-Ala-D-isoGlu-meso-DAP) have been demonstrated (Laroui et al., 2011). Notably, in their assay, the NOD1 ligand TriDAP bound the NOD1 LRR domain with a KD of 34.5 μM, which raises questions about the physiological relevance of TriDAP.

But how is ligand binding triggering the assembly of the NOD signaling complex? It was difficult to draw conclusions about the mode of action of signaling complex assembly from early models of NOD1 and NOD2, which were based on the crystal structures of homologous receptors such as apoptotic protease-activating factor 1 (Apaf-1) (Riedl et al., 2005; Proell et al., 2008). The recently published crystal structure of NOD2 provides a more detailed view on how structural changes impact on ligand binding and signal transduction. Under steady-state conditions, NOD2 remains in an inactive, closed conformation with tightly packed subdomains by ADP-mediated inter-domain interactions (Maekawa et al., 2016). Ligand binding to the LRRs and the exchange of ADP for ATP triggers the unfolding of the protein and stabilizes it in an active conformation (Maekawa et al., 2016). NOD2 oligomerizes via its NOD and CARD domains and recruits RIPK2 to form a hetero-CARD complex (Kobayashi et al., 2002; Fridh and Rittinger, 2012). Recent work showed that multiple RIPK2 monomers can then bind via homotypic CARD-CARD interactions to form fibrillar protein assemblies, termed higher-order signalosomes (Gong et al., 2018; Pellegrini et al., 2018). Single amino acid mutations in the CARD domain of RIPK2 that disrupt its oligomerization shut down MDP-dependent NF-κB responses (Pellegrini et al., 2018). Similar structures have been reported in other innate immune signaling pathways such as in the NLRP3, ASC, caspase-1 inflammasome (Lu et al., 2014) and are believed to facilitate and regulate signal transduction (Wu, 2013).

### Non-canonical Activation of NOD2

While activation of the NOD pathways through PGN stimulation is well documented, there are more recent reports of activation through PGN-independent pathways. Keestra-Gounder et al. (2016) observed that systemic pro-inflammatory responses triggered by thapsigargin and by infections with the ER-stress-inducing bacterium *Brucella abortus* are blunted in NOD1/2-deficient mice. The underlying mechanisms are still largely unclear, however, experiments conducted with a dominant-negative form of TRAF2 suggested that this process is TRAF2-dependent. The ER stress sensor IRE1 and TRAF2 have been previously shown to interact in overexpression studies and in yeast-two-hybrid screenings (Urano et al., 2000) and this interaction could link NF-κB and MAPK activation with stress pathways (Kaneko et al., 2003). Furthermore, earlier studies suggested that members of the TRAF family interact with the adaptor protein RIPK2, which functions downstream of NOD1 and NOD2 activation (Thome et al., 1998). NOD2 contains a predicted TRAF2-binding motif in its nucleotide-binding oligomerization domain (Schneider et al., 2012) and could therefore function as the link between ER stress and inflammatory signaling. A recent study confirmed that thapsigargin induces NOD-dependent pro-inflammatory signaling, although this was due to the compound’s inhibition of the sarcoplasmic or endoplasmic reticulum calcium ATPase family (SERCA), which is responsible for Ca^2+^ movement into the ER and cellular Ca^2+^ regulation (Molinaro et al., 2019). Thapsigargin-induced depletion of Ca^2+^ within the ER led to a rise in intracellular Ca^2+^ levels and enhanced both Ca^2+^ internalization and endocytosis. This endocytosis led to internalization of trace peptidoglycan contaminants in the cell culture grade FCS, which was confirmed using mass spectrometry.

Several pathogenic scenarios also point toward PGN-independent activation of the NOD pathway. Neuropathic pain, mediated by an inflammatory reaction of peripheral macrophages in mice that underwent nerve injury, results in the activation of the NOD2 pathway without the evident involvement of bacterial components (Santa-Cecilia et al., 2019). Furthermore, increased levels of phosphorylated RIPK2, a hallmark of NOD1/2 pathway activation, has been detected in neoplastic tissue of triple-negative breast cancer patients (Mertins et al., 2016). Also in this scenario, it is not directly evident that NOD pathway activation is a direct result of bacterial components and it will be interesting to further investigate the mode of activation in these tissues. It has to be noted though that secreted bacterial components such as OMVs could well be the underlying factor for these apparently non-canonical forms of activation of the NOD pathway.

### Signaling Downstream of NOD2 Activation

**FIGURE 2:**
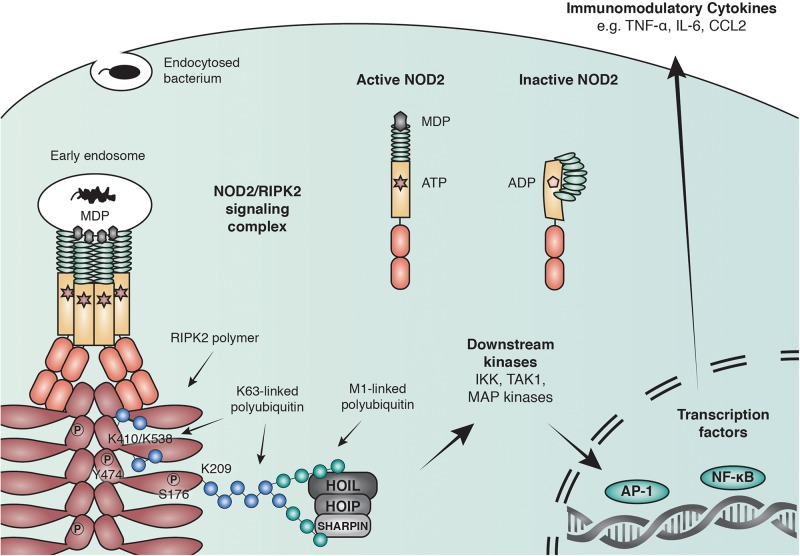
Anti-bacterial signaling mediated by NOD2. Within the family of NLRs, NOD2 represents a particularly well-studied receptor that is activated by binding to the peptidoglycan fragment MDP in the cytosol and at endosomal membranes. NOD2 recruits the adaptor molecule receptor-interacting serine/threonine-protein kinase 2 (RIPK2) through CARD-CARD interactions to form large polymers that facilitate the activation of downstream kinases and lead to the initiation of immune modulatory transcriptional responses through AP-1 and NF-κB transcription factors. RIPK2 is regulated through polyubiquitination by multiple E3 ubiquitin ligases including X-linked inhibitor of apoptosis protein (XIAP) and the linear ubiquitin chain assembly complex (LUBAC) and by phosphorylation of serine and tyrosine residues.

Binding of PGN to NOD1/2 and subsequent recruitment of RIPK2 results in the ubiquitination of RIPK2 and the recruitment of downstream effector proteins including the IKK complex and TAK1 (Park et al., 2007a; Kim J.Y. et al., 2008; Figure 2). The exact role of ubiquitin ligases and the consequence of RIPK2 ubiquitination will be discussed later. RIPK2 ubiquitination ultimately leads to the activation of key transcription factors such as NF-κB and AP-1. Synchronized activation of both transcription factors is required for the transcriptional response, as interference with the timing of activation using a RIPK2 inhibitor resulted in a reduced cytokine response (Nachbur et al., 2015).

Among the strongest induced genes downstream of NOD activation are immunomodulatory cytokines, such as TNF, IL-1β, IL-6, and CC-chemokine ligand 2 (CCL2/MCP-1) (Kobayashi et al., 2005; Gilmore, 2006; Conforti-Andreoni et al., 2010). Transcriptomic profiling of MDP stimulated macrophages revealed a specific gene set downstream of NOD2 compared to general inflammatory stimuli (Tigno-Aranjuez et al., 2014). Members of this set are preferentially involved in immune functions, nucleotide regulation, and cell metabolism. In endothelial cells and Langerhans cells, stimulation with MDP resulted in enhanced IL-6 production and the Th17-differentiation of T cells within the skin (Manni et al., 2011), suggesting that the transcriptional response downstream of NOD stimulation varies considerably between cell types.

While the major outcomes of the NOD1 and NOD2 pathway primarily occur via the activation of NF-κB transcription factors and cytokine production, NOD1 and NOD2 activation can also lead to autophagy induction to clear a bacterial threat. This is in line with NOD2 localization at early endosomes, and the role of NOD1/2 in intestinal homeostasis. Activated NOD has been shown to interact with the autophagy protein ATG16L1 at the site of bacterial entry, although whether RIPK2 is involved in this process is under debate. Two studies show the involvement of RIPK2 in autophagy induction (Cooney et al., 2010; Homer et al., 2010), while a third study observed autophagy induction occurring in the absence of RIPK2 (Travassos et al., 2010). However, the studies agree on the observation that autophagy induction is independent of NF-κB, using both infection models as well as purified ligands. Induction of autophagy downstream of NOD activation can have implications in several pathological conditions, particularly in Crohn’s diseases where mutations in the autophagy protein ATG16L1 are among the highest genetic risk factors to develop the disease.

## Ripk2, the Mediator of NOD Signaling

### RIPK2 Is a Member of the RIP Kinase Family

As mentioned above, RIPK2 is the central adaptor kinase in the NOD pathway. RIP kinases represent a class of serine/threonine kinases that play essential roles in the regulation of innate immune signaling. Their functions depend on the highly conserved N-terminal kinase domains and distinct C-terminal interaction motifs. Amongst the RIP kinases, RIPK1 and RIPK3 are the best-characterized members, which are being extensively studied due to their involvement in cell death and their role in chronic diseases and cancer. RIPK1 contains a C-terminal death domain (DD) that mediates direct binding to death receptors of the TNF receptor superfamily members including TNFR1, Fas, and TRAIL, and to adaptor proteins such as FADD or TRADD. Upon binding, oligomeric protein complexes are formed that can regulate survival or cell death. Under specific conditions, RIPK1 associates with RIPK3 through their RIP homotypic interaction motif (RHIM) to activate the pseudokinase mixed lineage kinase domain-like (MLKL) to induce necroptosis, a pro-inflammatory form of programmed cell death (Silke et al., 2015).

Receptor-interacting serine/threonine-protein kinase 2 (RIPK2) represents the next best-characterized member of the RIP kinase family. Compared to RIPK1 and RIPK3, RIPK2 does not have a RHIM or a DD and is therefore unable to interact with these death receptor complexes. RIPK2 is composed of an N-terminal kinase domain, an intermediate domain of unknown function, and a C-terminal CARD that mediates binding to NOD1/2 via homotypic CARD-CARD interactions (Inohara et al., 1998; McCarthy et al., 1998; Thome et al., 1998). Structural data of the kinase domain (Canning et al., 2015; He et al., 2017; Hrdinka et al., 2018; Suebsuwong et al., 2018) and the CARD of RIPK2 (Lin et al., 2015; Goncharuk et al., 2018) have recently been revealed. The kinase domain shows a typical kinase fold with the catalytic center located between a smaller N- and a larger C-lobe. The C-terminal CARD of RIPK2 displays typical features of a protein from the death domain family, but unlike other CARDs or death domains, the CARD of RIPK2 contains an additional sixth helix. The intermediate domain of RIPK2 is thought to be unstructured and highly flexible, however, posttranslational modifications in this domain upon stimulation could suggest so far unappreciated involvement in RIPK2s signaling function.

RIPK2 is indispensable for NOD-mediated activation of the NF-κB and MAPK pathways and its recruitment to NOD2 occurs via CARD-CARD interaction (Girardin et al., 2001; Park et al., 2007a; Magalhaes et al., 2011). An acidic patch in the NOD1 CARD forms the primary binding interface with basic residues in the RIPK2 CARD. Using mutational analysis and pulldown experiments, Manon et al. (2007) identified three acidic residues (E53, D54, E56) in helix 3 of the NOD1 CARD and three basic residues (R444, R483, R488) in the RIPK2 CARD as the key mediators of the NOD1-RIPK2 interaction. A more recent study proposed that two additional residues in RIPK2 (K443, Y474) are required for NOD1 binding (Mayle et al., 2014).

The NOD2-RIPK2 interface differs from that between NOD1 and RIPK2. Overexpression of both NOD2 CARDs is required for a constitutive NF-κB response (Ogura et al., 2001b). Even though the two CARDs of NOD2 do not act independently, the N-terminal NOD2 CARD (CARDa) is solely responsible for binding to RIPK2 (Fridh and Rittinger, 2012). In contrast to the interaction between NOD1 and RIPK2, the NOD2-RIPK2 interaction motif is made of two basic residues in the NOD2 CARDa (R38, R86) and a set of acidic residues on the RIPK2 CARD (D461, E472, D473, E475 and D492). Intriguingly, direct interaction between NOD2 and RIPK2 has so far only been described using recombinant proteins or in overexpression experiments, which suggests that under physiological conditions the NOD-RIPK2 interaction is either highly transient or unstable.

### Structure and Function of RIPK2

RIPK2 was originally identified as a serine-threonine kinase based on sequence homology (Inohara et al., 1998; McCarthy et al., 1998; Thome et al., 1998), but was later reclassified as a dual-specificity kinase that is also able to phosphorylate tyrosine residues (Tigno-Aranjuez et al., 2010). Although the importance of RIPK2 as the central player in NOD signaling has been demonstrated, the function of its kinase activity is still under debate. The active state of the kinase domain is dictated by an invariant lysine within the N-lobe (K47), which contacts and supports ATP. This interaction is supported by the formation of a salt bridge within the middle of the αC-helix (Kornev and Taylor, 2010).

The only substrate of RIPK2 that has been described so far, is RIPK2 itself. Upon activation by dimerization via the CARD domains of NOD1/2, RIPK2 autophosphorylates on S176 in the activation loop of the kinase domain (Dorsch et al., 2006) and on Y474 in its CARD (Tigno-Aranjuez et al., 2010). In overexpression systems, mutation of either of those residues decreased RIPK2’s ability to induce downstream signaling.

By comparing the phosphorylation profiles of wild-type RIPK2 vs. catalytically inactive mutants (K47A and D164N), it was observed that besides S176, multiple additional serine residues within the activation loop can be phosphorylated (Pellegrini et al., 2017). More phosphorylated residues have been discovered in large-scale proteomic screenings, however, their functional relevance remains to be evaluated (Daub et al., 2008; Oppermann et al., 2009). *In vitro* auto-phosphorylation assays indicated that catalytically inactive mutants could still be phosphorylated by purified full-length RIPK2, suggesting that autophosphorylation occurs in *trans* (Pellegrini et al., 2017), which requires strong interactions between two or multiple RIPK2 molecules. In line with this theory, biophysical measurements suggested that the active state RIPK2 is a stable dimer whilst the inactive kinase is in a monomer-dimer equilibrium. Supporting this, recently published crystal structures display the phosphorylated form of RIPK2 as a side-by-side dimer, suggesting that dimerization plays a critical role in kinase activation (Tigno-Aranjuez et al., 2010; Tigno-Aranjuez et al., 2014; Canning et al., 2015; Charnley et al., 2015; Nachbur et al., 2015; Haile et al., 2016).

While the ability of phosphorylation by RIPK2 was clearly demonstrated, it is still under debate whether the kinase function is required for NOD signaling. On the one hand side, studies utilizing overexpression of RIPK2 suggested that the kinase activity of RIPK2 is dispensable for NF-κB activation and cytokine production altogether. The catalytically dead mutants of RIPK2 (K47A and D146N) could still activate NF-κB signaling, although this occurred independently of NOD2 engagement with MDP (Inohara et al., 1998; Thome et al., 1998; Eickhoff et al., 2004; Hrdinka et al., 2018). On the other hand side, bone marrow-derived macrophages (BMDMs) from a kinase-dead (K47A) knock-in mouse were defective in signaling (Nembrini et al., 2009). However, kinase-dead RIPK2 was only expressed at very low levels, which could be the reason for deficient NOD signaling in this system and suggests that RIPK2’s kinase activity is required for protein stability rather than being an intrinsic requirement for NOD signaling. Recent studies also re-raised questions about the importance of the regulatory autophosphorylation sites S176 and Y474. Overexpression of RIPK2 mutants in HeLa cells showed that wild-type RIPK2 and the S176A mutant resulted in similar amounts of cytokines following infections with *S. flexneri*, while the S176E mutant resulted in reduced cytokine levels (Ellwanger et al., 2019). In contrast, cytokine production was completely abolished in cells expressing the RIPK2 Y474F mutant. The importance of Y474 for signal transduction was also highlighted in two recent studies that utilized cryo-EM to solve RIPK2 structures. Y474 was found to sit at a critical interface in the CARD and to mediate intermolecular interactions during RIPK2 polymerization, which was shown to be essential for NF-κB activation. Thus, it is not surprising and explains that a tyrosine to phenylalanine mutation disrupts RIPK2 activity (Gong et al., 2018; Pellegrini et al., 2018).

### RIPK2 Ubiquitination and Scaffolding

While the importance of the kinase activity of RIPK2 remains somewhat dubious, it has become clear that its ubiquitination is a critical determinant of downstream signaling. Ubiquitin is a small, 8.5 kDa protein that can be covalently attached via its C-terminus to lysine residues of target proteins or to the N-terminus of one of the seven lysine residues of a substrate-attached ubiquitin. The diverse biological outcomes of protein ubiquitination are dependent on the complex interplay between the sites of the ubiquitination, chain length, chain type, chain branching as well as posttranslational modifications on ubiquitin itself (Komander and Rape, 2012; Swatek and Komander, 2016).

Within the NOD signaling pathway, RIPK2 is the key substrate for this process. Upon NOD activation, multiple ubiquitination events have been described on RIPK2, which are required to induce the activation of NF-κB and MAPK pathways. Ubiquitination was first observed to regulate the NOD1 and NOD2 pathways in studies that utilized over-expression of RIPK2 and *Mycobacterium tuberculosis* infections in macrophages. RIPK2 was stably ubiquitinated, and this ubiquitination was required for optimal cytokine production (Hasegawa et al., 2008). These results led to several subsequent studies and today it is widely accepted that polyubiquitin chains on RIPK2 serve as binding platforms for downstream signaling proteins that are vital for the activation of NF-κB and MAP kinases. The key events downstream of RIPK2 ubiquitination are the recruitment of the NF-κB-activating IkB kinase (IKK) complex composed of IKKα, IKKβ and NEMO (Inohara et al., 2000; Yang et al., 2007; Hasegawa et al., 2008), TGF-β activated kinase (TAK1), which is recruited via the two ubiquitin-binding scaffold proteins MAP3K7-binding protein 2 and 3 (TAB 2 and 3) (Kanayama et al., 2004) and the linear ubiquitin chain assembly complex (LUBAC), which is composed of a catalytic subunit HOIP and the two regulatory subunits HOIL-1 and SHARPIN (Gerlach et al., 2011).

### IAPs: Critical E3 Ligases of RIPK2

**FIGURE 3:**
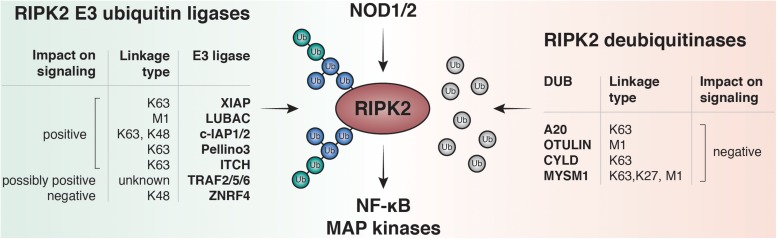
RIPK2 ubiquitination regulates NF-κB and MAPK activation by NOD1 and NOD2. Upon ligand binding to NOD1 or NOD2, RIPK2 is rapidly ubiquitinated with K63- and M1-linked polyubiquitin chains. The K63-specific E3-ligase XIAP and the Linear-Ubiquitin Assembly Complex (LUBAC) have been shown to be essential for downstream responses including activation of NF-κB and MAPK pathways *in vitro* and to induce robust PGN-dependent immune responses *in vivo*. Other E3 ligases are able to bind and ubiquitinate RIPK2 such as cellular inhibitor of apoptosis protein-1 and protein-2 (c-IAP1 and c-IAP2), pellino3, itchy E3 ubiquitin protein ligase (ITCH), TNF receptor associated factor 2, 5, and 6 (TRAF2, TRAF5, and TRAF6) and zinc and ring finger 4 (ZNRF4). Deubiquitinases negatively regulate NOD signaling by removing ubiquitin from RIPK2. Amongst them are A20, OTU deubiquitinase with linear linkage specificity (OTULIN), ubiquitin carboxyl-terminal hydrolase CYLD, and histone H2A deubiquitinase MYSM1.

So far K48, K63, M1 and more recently K27 ubiquitin linkages have been reported on RIPK2 (Damgaard et al., 2012; Panda and Gekara, 2018). Accordingly, an increasing number of E3 ligases and DUBs have been described to regulate the RIPK2 ubiquitin network (Figure 3). Screenings for ubiquitinated lysines within the kinase domain of RIPK2 suggested that ubiquitination of K209 is essential for signaling, and a RIPK2 K209R mutant was unable to activate NF-κB (Hasegawa et al., 2008).

A critical family of E3 ligases regulating NOD signaling are the IAPs. Cellular IAP1 and -2 (cIAP1/cIAP2), as well as X-linked IAP (XIAP), have all been reported to regulate NOD signaling (Bertrand et al., 2009; Krieg et al., 2009). The IAPs represent a group of cell death regulators and were initially described as caspase inhibitors, however, only XIAP is able to inhibit caspases at physiologically relevant concentrations. cIAPs, in turn, regulate cell death indirectly via their E3 ligase activity (Yang and Li, 2000). IAPs are defined by the presence of up to three approximately 70 amino acids long motifs called baculoviral IAP repeats (BIRs) (Birnbaum et al., 1994), which can mediate protein-protein interactions. Moreover, cIAP1, cIAP2, and XIAP contain a ubiquitin-associated domain (UBA) for binding to polyubiquitin chains and a really interesting new gene (RING) domain that provides E3 ligase activity (Silke and Vucic, 2014). The role of cIAP1 and cIAP2 in regulating TNF receptor signaling complexes is well-established: cIAPs directly ubiquitylate RIPK1 to facilitate activation of MAPK and canonical NF-κB pathways (Mahoney et al., 2008; Varfolomeev et al., 2008).

The first evidence that the cIAPs play a role in NOD signaling occurred in 1998. In HEK-293T cells overexpressed cIAPs co-immunoprecipitated with overexpressed RIPK2 (Thome et al., 1998). Bertrand et al. (2009) later showed that mice deficient in cIAP1 and cIAP2 had significantly reduced cytokine production in response to MDP injection compared to wild-type mice. Overexpression and pulldown experiments in HEK293T cells also suggested that cIAP1 and cIAP2 bind to and ubiquitinate RIPK2 independently of their CARD domains. However the role of the cIAPs in NOD signaling is controversial and we and other groups subsequently observed that removal of cIAP1/2 had no significant impact on signaling immediately downstream of NOD2 (Damgaard et al., 2012, 2013; Stafford et al., 2018). The discrepancy between the blunted cytokine response to MDP *in vivo* in cIAP1 and cIAP2-deficient mice but normal signaling in *ex vivo* stimulated BMDMs can be explained by a secondary autocrine loop that drives cIAP-dependent NF-κB and MAPK activation through TNFR1 (Stafford et al., 2018).

While recent studies argue against a critical role of cIAPs in NOD signaling, XIAP has emerged as a critical mediator of RIPK2 ubiquitylation and NOD signaling. The addition of K63-linked ubiquitin chains on RIPK2 is dependent on XIAP (Krieg et al., 2009; Damgaard et al., 2012). Using mouse and human cell lines devoid of XIAP, it was shown that XIAP is an indispensable component of the NOD signaling pathway and is required for the majority of ubiquitination on RIPK2. SPR recently revealed a direct interaction between the RIPK2 kinase domain and the BIR2 of XIAP (Goncharov et al., 2018). Consistently, IAP antagonists specifically targeting XIAP’s BIR2 disrupted this interaction, and impair RIPK2 polyubiquitination and downstream activation of MAPK and NF-κB pathways (Goncharov et al., 2018; Hrdinka et al., 2018). Adding to the first discovered ubiquitination site K209, Goncharov et al. also described further XIAP-dependent ubiquitination sites (K410/K538) on RIPK2 that, when mutated, reduce NF-κB activation and cytokine production.

### Other E3 Ligases and Deubiquitinases in the NOD Pathway

Ubiquitination of RIPK2 by XIAP is a vital step for subsequent recruitment of LUBAC (Damgaard et al., 2012), which is the only protein complex described so far to have the ability to add linear ubiquitin chains to substrates (Fiil et al., 2013; Tokunaga, 2013). It is not entirely clear whether linear ubiquitin chains are added on a previously non-ubiquitinated lysine residue of RIPK2, or as branching on a pre-existing ubiquitin chain. Cells lacking LUBAC subunits fail to fully activate NF-κB, which highlights the importance of LUBAC for efficient NF-κB and MAPK activation after NOD2 stimulation, possibly by recruiting and facilitating activation of the IKK complex.

Additional E3 ligases that mediate ubiquitination of RIPK2 to positively regulate NOD signaling have been reported: The TNF Receptor Associated Factors (TRAF) -2, -5, and -6, which are key adaptors in the TNFR1 signaling pathway contain a RING domain with E3 ligase activity. All three of these proteins have been linked to NOD signaling (Xie, 2013). So far there is no evidence for E3 ligase activity of TRAF2 and TRAF5 during NOD signaling, however, TRAF6 has been reported to directly contribute to RIPK2 ubiquitination. The knockdown of TRAF6 by siRNA in HEK293T cells reduced ubiquitination of RIPK2 and the induction of NF-κB following NOD2 stimulation (Yang et al., 2007). In another study, TRAF6 was not required for NOD signaling since TRAF6-deficient mouse embryonic fibroblasts (MEFs) still activated NF-κB and MAP kinases in response to NOD1 agonists (Hasegawa et al., 2008).

The E3 ligase Pellino3 was identified as another positive regulator of the NOD2 pathway, by mediating K63-linked ubiquitination of RIPK2. BMDMs from Pellino3-deficient mice displayed a lower activation of NF-κB and MAPK pathways and produce fewer cytokines after stimulation with MDP (Yang et al., 2013). Of note, the authors found a lower expression of Pellino3 protein in the colons of patients with Crohn’s disease, supporting the theory that Pellino3 is an important mediator of NOD2 signaling in the gut.

E3 ubiquitin-protein ligase Itchy homolog (ITCH) was also reported to be a direct E3 ligase for RIPK2, by adding K63-linked ubiquitin chains in *in vitro* ubiquitination assays and pulldown experiments (Tao et al., 2009). BMDMs from ITCH knock-out mice failed to ubiquitinate RIPK2 and had reduced activation of NF-κB and MAPK pathways and consequently reduced expression of NF-κB target genes after MDP-stimulation.

More recently, ZNRF4 was identified as a negative regulator of NOD2-dependent NF-κB activation in a genome-wide RNAi screening in HEK293T cells. ZNRF4 induced K48-linked ubiquitination of RIPK2 and promoted RIPK2 degradation. Moreover, ZNRF4 knockdown macrophages produced higher amounts of pro-inflammatory cytokines in response to MDP and ZNRF4 knockdown mice displayed reduced tolerance to secondary exposure to MDP and *L. monocytogenes* (Bist et al., 2017). To sum up, these data suggest that ZNFR4 could be part of a negative feedback loop to turn off prolonged and aberrant NOD2 signaling after pathway activation.

The removal of ubiquitin by linkage-specific DUBS fine-tunes NOD1 and NOD2 signaling. A20 was the first DUB identified to negatively regulate NOD2 signaling by cleaving non-K48-linked ubiquitin chains (Hitotsumatsu et al., 2008). OTULIN was shown to limit M1-linked ubiquitination of RIPK2 and antagonize LUBAC after NOD2 activation and subsequent NF-κB and MAPK signaling (Fiil et al., 2013). The ubiquitin carboxyl-terminal hydrolase CYLD targets both M1- and K63-linked ubiquitin linkages to limit NOD2 signaling (Hrdinka et al., 2016). Panda et al. showed that RIPK2 is also ubiquitinated with atypical K27-linked chains and Histone H2A deubiquitinase MYSM1 is a DUB that specifically removes K27-, K63- and M1-specific chains to dampen NOD2 signaling. Supporting a role in NOD signaling, MYSM1-deficient mice injected intraperitoneally with MDP exhibited higher recruitment of neutrophils to the peritoneum and peripheral organs (Panda and Gekara, 2018).

## Signaling Outcomes of NOD Activation

### NOD2 Signaling and Disease

Inflammatory bowel disease, particularly Crohn’s disease, is the most commonly associated pathology associated with NOD2 signaling (Caruso et al., 2014; Philpott et al., 2014). However there is compelling evidence that deregulated NOD1/2 signaling is associated with inflammation-associated diseases such as early-onset sarcoidosis, uveitis, neuropathic pain, rheumatoid arthritis or solid cancers (Caruso et al., 2014; Kim et al., 2016) and more recently with allergic asthma (Miller et al., 2018) and type 2 diabetes mellitus (T2DM) (Amar et al., 2011; Schertzer et al., 2011; Denou et al., 2015; Cavallari et al., 2017). Most of these disease associations have been reviewed extensively elsewhere (Kanneganti et al., 2007; Chen et al., 2009; Philpott et al., 2014; Mukherjee et al., 2019), and we focus here briefly on the most recent understanding of how NOD signaling can contribute to IBD or T2DM.

A clear hot spot for NOD2 related pathologies is the intestinal tract. The two key players in NOD2 signaling, NOD2 and RIPK2 are both highly expressed in intestinal epithelial cells as well as in resident immune cells in the gut. NOD2 seems to have an important role in gut homeostasis as there is evidence that NOD2 directly regulates colonic epithelial cell growth and survival. Nevertheless, NOD2-deficient mice do not have intestinal inflammation and display normal myeloid and lymphoid cellularity in the gut, at least in the absence of stimulation (Kobayashi et al., 2005). However NOD2-deficient mice do have reduced clearance upon oral or intragastric bacterial challenge (Kim et al., 2011). *In vitro*, primary colonic epithelial cells induced cell death in response to treatment with the NOD2 ligand MDP, while cells from NOD2-deficient mice were protected and shRNA-mediated knockdown of NOD2 in human colonic carcinoma cells resulted in increased levels of apoptosis (Cruickshank et al., 2008).

Several studies show an intimate link between NOD signaling and TLR signaling in the gut: NOD2 can significantly inhibit TLR4 signaling in enterocytes of the neonatal small intestine resulting in marked protection from the induction of TLR4-dependent apoptosis (Richardson et al., 2010). Furthermore, NOD2-deficient mice have exacerbated antigen-specific colitis that is dependent on TLR2 function (Watanabe et al., 2006). Subsequently it was shown that NOD2 protects in mouse models of experimental colitis via a cross-tolerance mechanism that dampens TLR responses (Hedl et al., 2007; Watanabe et al., 2008; Hedl and Abraham, 2011b), which relies on the induction of interferon regulatory factor 4 (Watanabe et al., 2014).

In experimental models of type 2 diabetes mellitus (T2DM), alterations in the intestinal barrier lead to increased intestinal permeability and translocation of PAMPs to the bloodstream, a phenomenon named metabolic endotoxemia (Cani et al., 2007). It is a well-established concept, that chronic exposure to low levels of bacterial components in the plasma, such as LPS or MDP, promotes inflammation and contributes to the development of hepatic insulin resistance. Therefore, it is not surprising that NOD1 and NOD2 agonists have been identified as modulators of insulin sensitivity. Intriguingly, the activation of either NOD1 or NOD2 leads to different outcomes in mouse models of T2DM: NOD1/2 double-knockout mice (Schertzer et al., 2011) and NOD1 knockout mice (Amar et al., 2011) were protected from HFD-induced insulin resistance. This effect was due to the role of NOD within immune cells, as bone marrow chimeras using bone marrow from NOD1-deficient mice transplanted into wild-type mice were protected against glucose and insulin tolerance (Chan et al., 2017). Unlike NOD1-knockout mice, animals deficient in NOD2 showed no protection to insulin resistance during HFD and even had increased adipose tissue and liver inflammation as well as exacerbated insulin resistance (Denou et al., 2015). Accordingly, injections of mice with the NOD2 ligands MDP and Mifamurtide reduced insulin resistance in mouse models of HFD-induced obesity and insulin resistance after endotoxic shock, while the NOD1 ligand FK565 worsened glucose tolerance (Cavallari et al., 2017). This divergence between the roles of NOD1 and NOD2 could be explained by the differential tissue and cellular distributions of the receptors.

### Pharmacological Inhibition of the NOD2 Pathway

Given the involvement of NOD2 and RIPK2 in a range of diseases, inhibition of RIPK2 could have an application in inflammatory diseases driven by dysregulated NOD signaling pathways. Kinase inhibitors with significant activity toward RIPK2 are already approved for clinical use, such as the multi-tyrosine kinase inhibitor ponatinib and the EGFR inhibitor gefitinib (Canning et al., 2015). Over the last years, significant efforts have been put towards the development of more specific RIPK2 inhibitors and multiple compounds have been successfully tested in mice. Two groups independently developed highly specific RIPK2 inhibitors, that could efficiently block cytokine production *in vivo* after intraperitoneal administration of MDP (Goncharov et al., 2018; Hrdinka et al., 2018). Furthermore, a specific RIPK2 inhibitor WEHI-345, was used to protect against the onset of paralysis in the experimental autoimmune encephalomyelitis (EAE) model for multiple sclerosis (Nachbur et al., 2015). These experiments also showed that even though RIPK2 kinase inhibitors bind into the ATP-binding pocket and block its kinase activity, their real mode of action is by blocking NOD signaling through disruption of the RIPK2-XIAP interaction. Lastly, GlaxoSmithKline has tested their RIPK2 kinase inhibitor GSK-559 in Phase 1 clinical trials for IBD, however, they have recently terminated their RIPK2 program.

An alternative approach to inhibit the NOD pathway is to antagonize the critical E3 ligases IAPs. However, compounds that target cIAPs and XIAP are not tolerated in the clinic as they induce an inflammatory response *in vivo* (Lawlor et al., 2015). Until recently, all reported compounds with activity toward XIAP were pan IAP inhibitors (Condon et al., 2014). Recently new compounds that only target XIAP have been developed and could be promising tools to block NOD signaling without inducing cell death (Goncharov et al., 2018). Similar to RIPK2 inhibitors, these new compounds antagonize NOD signaling by disrupting the RIPK2-XIAP interaction.

## Is NOD Signaling Linked to Cell Death?

As discussed in detail above, signaling downstream of NOD1/2 harbors many proteins and protein domains that are closely associated with cell death signaling. A link between NOD signaling and cell death induction seemed therefore likely ever since NOD signaling was studied.

NOD1, NOD2, and RIPK2 harbor one or multiple highly conserved CARDs, which are known to recruit caspases, the key mediators of apoptosis. It is therefore not surprising that all these proteins have initially been associated with caspase binding and with programmed cell death. Indeed, overexpression studies with NOD2 showed that it could bind to multiple caspases via its CARD, and was able to directly activate caspase-9 and induce apoptosis (Inohara et al., 1999). This was attributed to the analogy to Apaf-1, the well-characterized activator of caspase-9 in the intrinsic apoptosis pathway. Similarly, NOD1 was able to directly activate Caspase-9 in a RIPK2-dependent manner. This was somewhat surprising as also RIPK2 was shown to interact with Caspase-9, but not to activate it. It was therefore suggested that RIPK2 needs to interact with NOD1 for caspase-9 activation (Bertin et al., 1999).

An indirect link between NOD signaling and apoptosis was suggested in early studies on RIPK2, which showed that overexpressed RIPK2 could potentiate CD95-induced apoptosis via caspase-8 and caspase-10 (Inohara et al., 1998). ATP binding to RIPK2 was critical for this function as the mutation of K38 resulted in reduced cell death after CD95L stimulation. Notably, RIPK2 also interacted with various members of the death receptor machinery, including cellular FLICE (FADD-like IL-1β-converting enzyme)-inhibitory protein (c-FLIP), cIAP1 and cIAP2 and members of the TNFR-associated factor (TRAF) family (Thome et al., 1998). These findings suggested that RIPK2 may play a role in the regulation of cell death, which was supported by experiments conducted in MCF-7 breast carcinoma cells, where overexpression of RIPK2 induced apoptosis (McCarthy et al., 1998). The cell death-inducing function of RIPK2 was dependent on the CARD and could be blocked with the caspase inhibitor zVAD.

The strongest evidence that suggests direct involvement of NOD signaling pathways in regulating caspase functions stem from observations that NOD1 and NOD2 can induce IL-1β through NF-κB and MAPK pathways in multiple human and mouse cell populations, including myeloid-derived cells (Li et al., 2004; Watanabe et al., 2004; Abraham and Cho, 2009). Moreover, there is evidence that NOD2 directly activates caspase-1 in certain cell lines (Damiano et al., 2004; Ferwerda et al., 2008; Hsu et al., 2008; Marina-Garcia et al., 2008). In human monocyte-derived macrophages (MDMs), activation of NOD2 leads to rapid IL-1β processing and autocrine signaling, a process that was essential for robust cytokine production (Hedl and Abraham, 2011a). The authors measured early MAPK activation, which was dramatically reduced by blocking IL-1β signaling and by inhibiting caspases using zVAD. Since the effects were visible already before transcription, translation, and secretion of IL-1β would be expected to occur, a model where NOD2 stimulation activates caspase-1, leading to the rapid processing of preformed pro-IL-1β, which in turn mediates early MAPK activation was suggested (Hedl and Abraham, 2011a).

A surprising finding was presented later, when it was shown that Bid, a well-characterized pro-apoptotic member of the Bcl2 family, was shown to be required for NOD signaling, as cells and mice deficient in Bid were not able to react to MDP (Yeretssian et al., 2011). However this finding was refuted shortly after (Nachbur et al., 2012) and Bid has since not been linked to NOD signaling, nor has it come up in screens for regulators of the NOD signaling pathway (Warner et al., 2014).

While there seems to be no direct link between NOD signaling and apoptosis, there is a strong link between NOD signaling and autophagy, the disassembly of damaged or unnecessary cellular components, that can result in death. In the context of NOD signaling, autophagy is more likely to be a cellular defense mechanism for bacterial clearance rather than a cell death mechanism.

Taken all together, initial experiments that linked NOD signaling with cell death could not be confirmed when endogenous protein levels and physiological ligands were used in later experiments. While overexpression studies are an important tool to determine molecular mechanisms of cell signaling, it has become clear that one has to be cautious when assessing the effects of overexpressed proteins on cell death. The last decade has seen many advances in establishing the links between innate immune signaling pathways and cell death, using mainly myeloid cells and relevant ligands. It has become clear that the link between NOD signaling and cell death is not as straight forward as initially thought, despite the indisputable involvement of cell death-related proteins and cell death-promoting domains.

## Conclusion and Perspectives

The title of this research topic is “Connecting the dots between inflammatory signaling and the working of cell death.” Here we have dissected the molecular mechanisms of signaling downstream of the intracellular PGN receptors NOD1/2. We have found that a critical point of difference between the NOD pathway and other innate immune signaling pathways is its failure to connect these dots. This is somewhat surprising. Not only do most inflammatory signaling pathways directly or indirectly induce cell death, but also have early studies implicated that activation of the NOD signaling pathway results in caspase activation and apoptosis. The development of new reagents and model systems has led to studies using endogenous proteins and specific means to stimulate the NOD pathways, as well as the use of relevant cell types. This is in contrast to earlier studies that were largely based on overexpression of members of the NOD pathway. In these newer work, the initial findings that NOD1/2 activation leads to any form of cell death could not be confirmed.

So what is different between the NOD pathway and other cell death-inducing inflammatory pathways? One reason could be that the NOD pathway is not exclusively pro-inflammatory at all. The best evidence is the strong association of NOD2 mutations with Crohn’s disease: These are prominently loss of function mutations within NOD2, suggesting that NOD signaling has an anti-inflammatory role. Conversely, hyperactivation of the NOD pathway is described in other inflammatory diseases and elevated RIPK2 activation, a hallmark of NOD signaling, is observed in many pathologies, intriguingly also in patients with IBD. Therefore, the NOD pathway rather plays an immunomodulatory role, rather than a pro-inflammatory. A cell that induces inflammation needs to be shut down rapidly to avoid hyperactivation of an inflammatory response, and a potent way to do so is to induce programmed cell death in this cell. If NOD signaling is, however, not as inflammatory at all, there is no need to self-destruct and hence the missing link between NOD signaling and cell death.

Despite the missing link between cell death and NOD signaling, this pathway has emerged as an important contributor to human pathologies. Therefore, significant efforts have been put toward better understanding the molecular mechanisms of NOD signaling. The focus for the development of therapeutics interfering with NOD signaling has been the kinase RIPK2, and several ATP competitive inhibitors have been developed by commercial and academic entities. The most recent data show convincingly, however, that the kinase activity of RIPK2 is dispensable for downstream signaling, and the critical role of RIPK2 is its scaffolding function in the pathway. Therefore, the understanding of protein-protein interactions and the ubiquitin network on RIPK2 and other members of the NOD pathway is pivotal for the development of novel therapeutics in this space.

## Author Contributions

VH generated the figures. VH, CS, and UN wrote the review.

## Conflict of Interest

The authors declare that the research was conducted in the absence of any commercial or financial relationships that could be construed as a potential conflict of interest.
